# APOE ε4 carriage is associated with olfactory-hippocampal tract functional connectivity

**DOI:** 10.1007/s11682-026-01109-x

**Published:** 2026-03-13

**Authors:** Toshikazu Ikuta, Taylor Bither, The Alzheimer’s Disease Neuroimaging Initiative

**Affiliations:** 1 Department of Communication Sciences and Disorders, School of Applied Sciences, University of Mississippi, PO Box 1848, University, MS 38677 USA; 2 School of Nursing and Health Studies, University of Miami, Coral Gables, FL 33124 USA

**Keywords:** APOE, Alzheimer’s disease, Olfaction, Resting state functional connectivity

## Abstract

Olfactory dysfunction often emerges before cognitive symptoms and may signal early vulnerability to neurodegenerative processes. This study examined whether genetic risk, specifically the presence of the epsilon 4 allele in apolipoprotein E, is associated with altered functional connectivity between the hippocampus and olfactory regions. Resting-state functional imaging data from 126 participants (mean age = 71.8 years, SD = 6.9; 67 females) across a range of clinical stages were analyzed. Functional connectivity was computed between the hippocampus and four olfactory-related regions: anterior piriform cortex, posterior piriform cortex, olfactory bulb, and olfactory tract. Multiple regression models assessed whether genetic risk, age, sex, and clinical diagnosis predicted connectivity strength. Genetic risk was significantly associated with increased connectivity between the hippocampus and the olfactory tract (model R² = 0.12). A nominal APOE ε4 effect was also observed in the olfactory bulb, although the overall model did not reach significance, while no significant effects were observed in the piriform cortex regions. Clinical diagnosis was not a significant predictor of connectivity in any region. These results suggest that genetic risk is linked to early functional reorganization in specific olfactory-hippocampal pathways, particularly the olfactory tract, independent of clinical progression. The olfactory-hippocampal network may serve as a sensitive target for detecting early brain changes associated with neurodegenerative risk.

## Introduction

The olfactory system is more sensitive to Alzheimer’s Disease (AD) than other parts of the brain. Olfactory dysfunctions are exhibited before cognitive and memory impairments (Wilson et al., 2009). This means that there are parts of the brain that are influenced prior to the onset of the noticeable symptoms. Olfactory deficits have been further found to predict later cognitive decline (Devanand et al., 2015). Smell identification has been suggested to be a useful screening tool for AD (Woodward et al., 2017).

Olfactory deficits and dysfunctions (Wesson et al., 2010) have both been found to be associated with beta-amyloid accumulations, which has been one of the most studied biomarkers so far. It is plausible that the same underlying pathology responsible for cognitive decline in Alzheimer’s disease also contributes to olfactory deficits.

The hippocampus is anatomically and functionally connected with key olfactory regions, including the piriform cortex, olfactory bulb, and olfactory tract, forming an integrated olfactory–limbic network involved in odor processing and memory (Gottfried et al., 2004; Shipley & Ennis, 1996). The hippocampus is considered to be responsible for memory and cognitive decline in AD (DeTure & Dickson, 2019; Halliday, 2017). Olfaction-Hippocampal functional connectivity was found to be affected in AD (Lu et al., 2019). Olfaction has long been implicated in its close association with the hippocampus (Eichenbaum & Otto, 1992). They are anatomically connected. Hippocampal CA1 region has been shown to have olfactory afferents (Biella & de Curtis, 2000). Recently, human olfactory loss has been found to reduce hippocampal activation in emotional memory (Han et al., 2019).

Olfactory dysfunction is not unique to AD. Olfactory deficits were found in some other disorders, such as Parkinson’s disease (R. L. Doty et al., 1988; Ross et al., 2008), and schizophrenia (Kästner et al., 2013; Moberg et al., 2014); and they have been found to be more prevalent in bipolar disorder, depression and autism (Hardy et al., 2012; Kamath et al., 2018; Taalman et al., 2017; Tonacci et al., 2017). Understanding olfactory networks in AD may allow us to deepen our knowledge about broader neurological mechanisms.

Genetic influence on olfaction is well documented. Olfactory function has been shown to be more sensitive to APOE ε4 carriage and neurodegenerative changes than memory or cognition (Bacon et al., 1998; Josefsson et al., 2017; Oleson & Murphy, 2015). The ɛ4 allele in Apolipoprotein E (APOE) is one of the strongest genetic risk factors for Alzheimer’s disease (Belloy et al., 2019). APOE genotype has also been associated with olfactory ability (Bacon et al., 1998; Woodward et al., 2017) with APOE ɛ4/ɛ4 homozygotes having greater olfactory dysfunction compared to their ɛ3/ɛ4 heterozygotes and ɛ3/ɛ3 homozygotes counterparts (Oleson & Murphy, 2015). This suggests that ɛ4 alleles influence olfaction. Heterozygotes of APOE ε4 showed olfactory decline in middle age adults, but did not show cognitive decline (Josefsson et al., 2017). It is implied that olfaction is more sensitive to APOE ε4 than cognition. Knockout of apoE showed olfactory deficiency in rodents (Nathan et al., 2004), further suggesting that the olfactory dysfunction may be a fundamental influence found also in rodents. More recent studies in humanized APOE mice also demonstrate that APOE impacts olfactory networks and brain connectivity, reinforcing its translational relevance (Moon et al., 2025; Stout et al., 2024). Understanding olfactory dysfunction may facilitate translating animal models into the human context. Despite clearly known associations between APOE ε4 and olfaction and between APOE ε4 and AD, the underlying mechanism between APOE genotype and AD has not been abundantly understood. Recent systematic and functional connectivity studies provide important context for olfactory network organization in humans (Bothwell et al., 2023; Zhou et al., 2019), demonstrating that olfactory networks represent a critical site of early functional change.

In this study, we aimed to evaluate whether olfactory functional connectivity may serve as a potential endophenotype of APOE ε4, providing an intermediate neural phenotype that links genetic risk with Alzheimer’s disease–related network vulnerability.

## Methods

Data used in this study were obtained from the Alzheimer’s Disease Neuroimaging Initiative (ADNI) database (adni.loni.usc.edu). ADNI was launched in 2003 as a public–private partnership led by Principal Investigator Michael W. Weiner, MD. The original aim of ADNI was to determine whether serial magnetic resonance imaging (MRI), positron emission tomography (PET), other biological markers, and clinical and neuropsychological assessments could be combined to measure the progression of mild cognitive impairment and early Alzheimer’s disease. Current objectives include validating biomarkers for clinical trials, increasing cohort diversity to improve generalizability, and providing open-access data to support research on the diagnosis and progression of Alzheimer’s disease. For the most up-to-date information, please visit *adni.loni.usc.edu*. Raw MRI files were downloaded as compressed DICOM images and subsequently converted into NIFTI format. APOE genotype was obtained from ADNIMERGE table. The APOE4 variable reflects the number of ε4 alleles (0, 1, or 2). Accordingly, the 0 group includes ε2/ε2, ε2/ε3, and ε3/ε3; the 1 group includes ε2/ε4 and ε3/ε4; and the 2 group corresponds to ε4/ε4.

A total of 126 participants were included in the analysis. The mean age was 71.8 years (SD = 6.9). The sample included 67 females (53.2%) and 59 males (46.8%). Baseline diagnostic categories were distributed as follows: 26 participants (20.6%) were diagnosed with Alzheimer’s disease (AD), 24 (19.0%) were cognitively normal (CN), 34 (27.0%) had early mild cognitive impairment (EMCI), 23 (18.3%) had late mild cognitive impairment (LMCI), and 19 (15.1%) were classified as having subjective memory complaints (SMC).

### Preprocessing

Initial data preprocessing followed the procedures outlined in a previous study (Kiparizoska & Ikuta, 2017). Data preprocessing and statistical analyses were conducted using the FMRIB Software Library (FSL) and the Analysis of Functional NeuroImages (AFNI). The anatomical volume for each subject was skull-stripped, segmented into gray matter, white matter, and cerebrospinal fluid (CSF), and registered to the MNI152 2 mm standard space. Through this registration process, 12 affine transformation parameters were generated to align the resting-state fMRI (rsfMRI) volumes with the MNI152 2 mm space, enabling subsequent registration of the processed EPI volumes.

ROI volumes were computed from the MNI 2 mm template-space masks using voxel count multiplied by voxel volume (8 mm³). The resulting template-space volumes were 240 mm³ for the anterior piriform cortex, 128 mm³ for the posterior piriform cortex, 160 mm³ for the olfactory bulb, and 192 mm³ for the olfactory tract. Because all olfactory ROIs were manually defined once in template space and applied identically to all subjects following registration, ROI volumes are invariant across participants.

The first four EPI volumes were discarded to allow signal stabilization. Transient signal spikes were removed using de-spiking interpolation. To correct for head motion, each volume was linearly registered to the first remaining volume, from which six motion parameters and the displacement distance between consecutive volumes were estimated. Each rsfMRI volume was then regressed using signals from white matter and CSF, along with the six motion parameters, to minimize physiological and motion-related noise.

Following regression, the data were smoothed using a 6 mm full-width at half-maximum (FWHM) Gaussian kernel, resampled, spatially transformed, and aligned to the MNI152 2 mm standard brain space. Motion scrubbing was conducted by calculating the root mean square (RMS) deviation of head displacement between successive volumes using a 40 mm radius spherical surface, as implemented in FSL’s rmsdiff tool (Power et al., 2015). Volumes exceeding a displacement threshold of 0.3 mm were excluded from further statistical analyses (Siegel et al., 2014).

### Registration of the olfactory regions

**Fig. 1:**
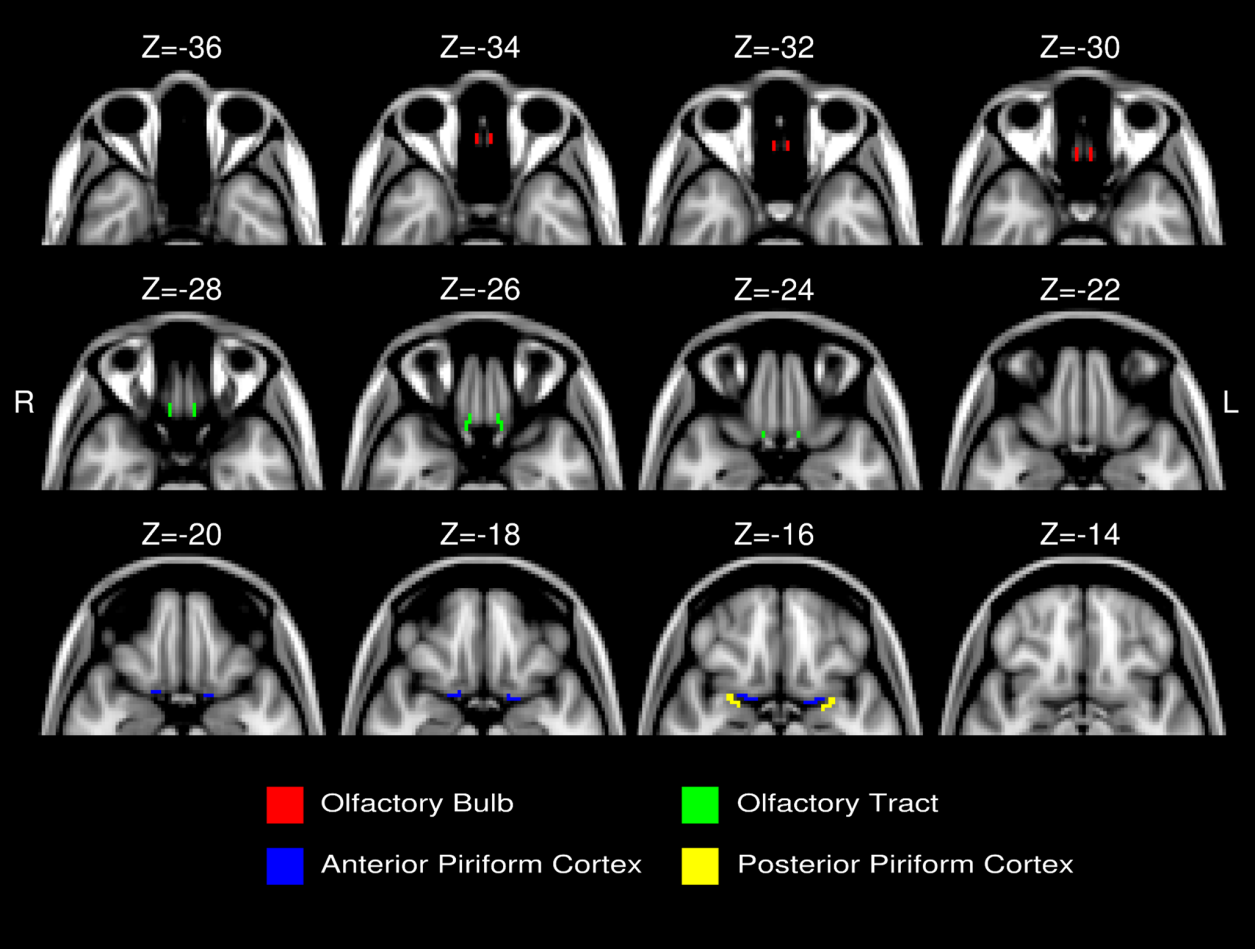
Four regions of interest (ROIs) specified in the MNI 152 2-mm brain space. Axial images shown included regions anterior to the brain stem (y ≧ −12), adapted from Kiparizoska and Ikuta (2017) licensed under CC BY-NC 4.0

Following the methodology established in a previous schizophrenia study (Kiparizoska & Ikuta, 2017), four regions of interest (ROIs); the olfactory bulb, olfactory tract, anterior piriform cortex, and posterior piriform cortex; were manually segmented in MNI (Montreal Neurological Institute) 2 mm space based on anatomical descriptions found in the literature (Gottfried, 2010; Howard et al., 2009; Scherfler et al., 2013). These olfactory ROIs were defined within the MNI 2 mm standard space. Representative overlays of these ROIs in MNI space are shown in Fig. 1 (adapted from Kiparizoska & Ikuta, 2017) to illustrate anatomical plausibility and registration quality.

Although accurate registration of individual functional datasets may result in proper alignment of the olfactory ROIs with their corresponding anatomical regions, all registrations were visually inspected in the MNI-registered anatomical space to ensure precision. Each individual session was evaluated following preprocessing by two authors (TB and TI), who independently inspected all 656 series while remaining blinded to each other’s assessments. The inspection involved verifying the alignment between the MNI-registered anatomical volumes and the MNI 2 mm template, as well as confirming the appropriate positioning of the four olfactory ROIs.

Each session was rated on a five-point scale ranging from 1 (significant problems) to 5 (no detectable issues). Upon completion of the visual inspection, evaluations from the two authors were compared by the principal investigator. Any series that received a score of 4 or lower (indicating any level of misalignment or issue) from both raters were excluded from further analysis.

### Connectivity analysis of the olfactory regions

The bilateral hippocampi were defined anatomically using the Harvard-Oxford Subcortical Structural Atlas. Resting-state functional connectivity was computed between the hippocampi and each of the four olfactory-related regions: the anterior piriform cortex (APC), posterior piriform cortex (Piri), olfactory bulb (OB), and olfactory tract (OT). ROI-to-ROI connectivity analyses were conducted at the individual subject level, resulting in Fisher’s *Z*-transformed correlation coefficients representing connectivity strength between the hippocampus and each olfactory region. These *Z*-scores were used as dependent variables in linear regression models. Multiple linear regression analyses were conducted to examine whether APOE ε4 allele count, age, sex, and baseline clinical diagnosis predicted hippocampal functional connectivity with four olfactory-related brain regions. These regression models were run separately for each olfactory region.

## Results

For the **anterior piriform cortex**, none of the predictors, including APOE ε4 (*p* =.608) and diagnosis (*p* =.911), were significantly associated with functional connectivity. The overall model was not significant, *F*(4, 120) = 0.09, *p* =.986, and explained minimal variance (*R*² = 0.003).

For the **posterior piriform cortex**, the model remained non-significant, *F*(4, 120) = 0.89, *p* =.471, with no significant effects for APOE ε4 (*p* =.203) or diagnosis (*p* =.408). Only the intercept reached significance (*p* =.048), suggesting baseline elevation in connectivity values but no meaningful modulation by predictors.

In contrast, for the **olfactory bulb**, APOE ε4 was significantly associated with increased hippocampal connectivity (*β* = 0.090, *p* =.026), whereas diagnosis again did not contribute significantly (*p* =.988). The overall model was not significant, *F*(4, 92) = 1.47, *p* =.218.

**Fig. 2:**
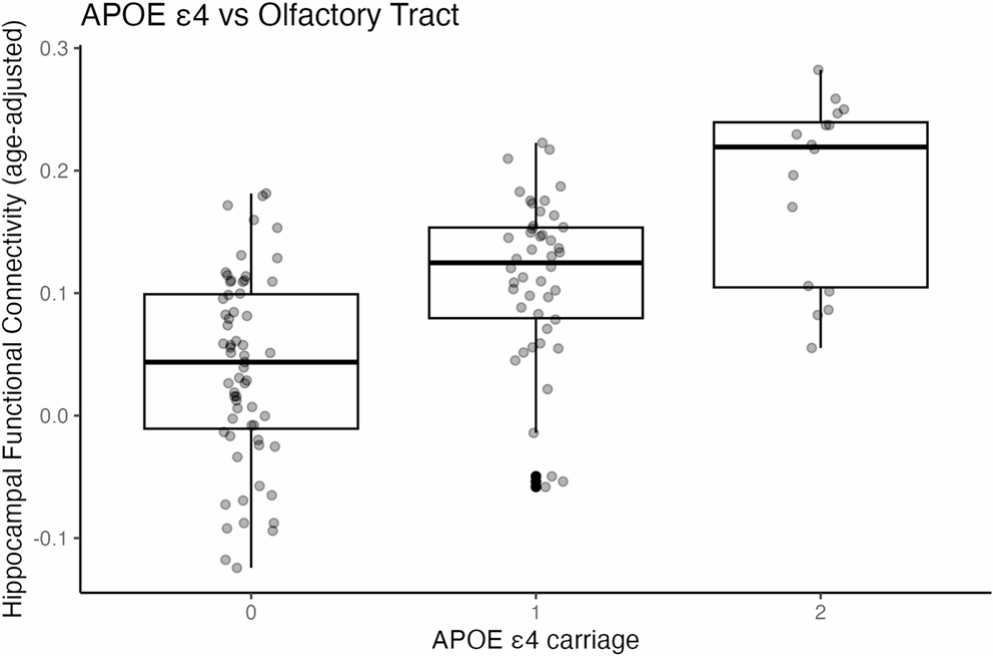
Association between APOE ε4 carriage and olfactory-hippocampal tract connectivity. Multiple linear regression controlling for age, sex, and baseline diagnosis showed a positive association between APOE ε4 allele count and olfactory-hippocampal tract connectivity (β = 0.080, *p* =.022). Age was also a significant predictor (β = 0.010, *p* =.003). The overall model was significant (F(4, 120) = 4.15, *p* =.004; FDR q = 0.016), explaining 12.1% of the variance (R² = 0.121). *n* = 125 for this analysis

For the **olfactory tract**, both APOE ε4 (β = 0.080, *p* =.022) and age (β = 0.010, *p* =.003) were significant predictors of increased connectivity. Diagnosis did not predict connectivity (*p* =.747). The model was statistically significant, *F*(4, 120) = 4.15, *p* =.004 (FDR q = 0.016), explaining 12.1% of the variance (*R*² = 0.121). Diagnosis did not predict connectivity (*p* =.747), and the APOE ε4 × diagnosis interaction was not significant. As illustrated in Fig. 2, APOE ε4 carriage was associated with increased olfactory-hippocampal tract connectivity (β = 0.080, *p* =.022; model R² = 0.121; *n* = 125), controlling for age, sex, and diagnosis.

## Discussion

In this study, we investigated whether APOE ε4 carriage is associated with hippocampal functional connectivity to four olfactory-related brain regions—anterior piriform cortex (APC), posterior piriform cortex (PPC), olfactory bulb (OB), and olfactory tract (OT)—after accounting for age, sex, and baseline clinical diagnosis. The results indicated that individuals with higher APOE ε4 allele count tend to show stronger functional connectivity between the hippocampus and the olfactory tract. In other words, APOE ε4 carriage is associated with increased communication or synchronization between these two brain regions, OT and hippocampus. Additionally, older age was independently associated with greater connectivity in the same pathway. Together, these factors explained about 12% of the variability in connectivity strength, suggesting that both genetic risk and age contribute meaningfully to differences in olfactory-hippocampal tract interactions. Although statistically robust, the effect size was modest (R² = 0.121); therefore, results should be interpreted cautiously in light of the cross-sectional design and possible unmeasured confounds.

Although APOE ε4 is associated with AD risk and olfactory decline, increased hippocampal–olfactory functional connectivity in APOE ε4 carriers is consistent with reports of early hyperconnectivity and hyperactivation in preclinical APOE ε4 populations, often interpreted as compensatory or dedifferentiated recruitment rather than improved function (Bookheimer et al., 2000; Filippini et al., 2009; Hodgetts et al., 2019). Mechanistically, APOE ε4 is linked to GABAergic interneuron vulnerability and network hyperexcitability (Andrews-Zwilling et al., 2010; Leung et al., 2012), which can increase low-frequency synchrony measured by BOLD. In parallel, APOE ε4-related neurovascular alterations (reduced or dysregulated neurovascular coupling and altered cerebrovascular reactivity) may further shape BOLD correlations (Koizumi et al., 2018; Pearson et al., 2024; Tai et al., 2016). Thus, stronger hippocampal coupling with the olfactory tract/bulb in APOE ε4/ε4 likely reflects early network reorganization and/or hyperexcitability rather than preserved integrity, aligning with models in which heightened connectivity precedes later decline. Consistent with this view, the APOE ε4 × diagnosis interaction was not significant, suggesting that APOE-related differences in olfactory-hippocampal connectivity may occur independently of clinical stage.

Although most functional connectivity studies focus on gray matter nodes, recent work demonstrates that BOLD signals in white matter are reliable and functionally meaningful. White matter BOLD fluctuations show spatially organized patterns, participate in resting-state networks, and relate to cognitive measures. Thus, including the olfactory tract is consistent with emerging evidence that tracts can contribute to functional network characterization. Regarding the olfactory bulb, while it is the primary receptor of olfactory input and temporal dynamics may differ from higher-order structures, our results emphasize network-level associations rather than direct timing of activation. Finally, we note that additional olfactory-related regions, such as the entorhinal cortex, amygdala, and olfactory tubercle, are also central to olfactory–limbic interactions (Courtiol & Wilson, 2017). These regions should be prioritized in future studies to expand the scope of olfactory–hippocampal network investigation.

Taken together, these findings are most consistent with early network vulnerability and preclinical change rather than direct clinical markers. The absence of associations with diagnosis suggests that APOE-related differences in olfactory-hippocampal connectivity may precede overt disease expression and could serve as indicators of genetic risk. Future longitudinal studies incorporating behavioral olfactory measures and additional biomarkers will be necessary to determine whether these connectivity differences predict subsequent decline or contribute to risk prediction models.

Although the association between APOE ε4 and connectivity in the olfactory bulb reached statistical significance, the lack of overall model significance limits the interpretability of this finding. Previous studies have shown mixed results regarding APOE ε4-related changes in olfactory bulb structure and function, with some reporting early degeneration (Wesson et al., 2010) and others noting region-specific variability (Murphy, 2019). Further investigation in larger or stratified samples will be necessary to determine whether the olfactory bulb reliably exhibits APOE ε4-related alterations in functional connectivity. The absence of significant findings in the APC and PPC regions aligns with prior work suggesting that piriform cortex involvement may occur later in the disease process (Jones et al., 2011; Sheline & Raichle, 2013) or may be less sensitive to early genetic risk factors. This pattern suggests that the effects of APOE ε4 are not uniformly distributed across the olfactory system.

Importantly, we found no significant associations between baseline clinical diagnosis and hippocampal connectivity in any of the four regions examined. This suggests that diagnosis, when modeled as an ordinal continuum from cognitively normal to Alzheimer’s disease, may not correspond linearly with functional alterations in the olfactory-hippocampal network. Prior functional connectivity studies have shown that such changes can precede clinical symptoms and may not correlate directly with diagnostic staging (Jones et al., 2011; Sheline & Raichle, 2013). It is possible that these connectivity differences occur independently of clinical stage or that categorical or nonlinear representations of diagnosis are more suitable (Greicius et al., 2004). The lack of a diagnostic effect also supports the use of functional connectivity as a potential early marker of neurodegenerative risk, particularly in genetically at-risk populations (Filippini et al., 2009). Finally, the APOE ε4 × diagnosis interaction was not significant, and our cross-sectional design limits inferences about stage-dependent effects.

This study has several limitations. First, the cross-sectional design limits our ability to infer the temporal progression of APOE ε4-related connectivity changes or their relationship to future cognitive decline. Longitudinal imaging studies will be necessary to determine whether these patterns predict clinical conversion or progression. Second, while we used an ordinal scale for diagnosis to reflect disease progression, categorical or biomarker-based classifications (e.g., amyloid/tau status) may provide more biologically relevant groupings (Jack Jr et al., 2018). Finally, our sample size—particularly in stratified diagnostic groups—may have reduced statistical power to detect subtle effects, especially in the piriform cortex, where signal variability is known to be high in functional imaging studies (Kopietz et al., 2009).

## Conclusions

These findings highlight the olfactory tract as a potentially sensitive site for APOE ε4-related functional changes. While the results in the olfactory bulb were strongly suggestive, only the olfactory tract showed a statistically robust association. Further research incorporating longitudinal data and additional biomarkers is needed to determine the temporal and clinical significance of these connectivity patterns.

## Data Availability

The original data is available at the ADNI website.

## Code availability

The functional connectivity codes will be available at https://olemiss.edu/projects/dnl/ upon publication.
